# A Syntenic Region Conserved from Fish to Mammalian X Chromosome

**DOI:** 10.1155/2014/873935

**Published:** 2014-11-18

**Authors:** Guijun Guan, Meisheng Yi, Tohru Kobayashi, Yunhan Hong, Yoshitaka Nagahama

**Affiliations:** ^1^Key Laboratory of Exploration and Utilization of Aquatic Genetic Resources, College of Fisheries and Life Sciences, Shanghai Ocean University and Laboratory of Reproductive Biology, Ministry of Education, Huchenghuan Road 999, Shanghai 201306, China; ^2^Laboratory of Reproduction, National Institute for Basic Biology, Okazaki, Aichi 444-8585, Japan; ^3^Laboratory of Molecular Reproductive Biology, School of Marine Sciences, Sun Yat-sen University, 135 Xingang West Road, Guangzhou, China; ^4^Lab of Molecular Developmental Biology, Institute for Environmental Sciences, University of Shizuoka, Yada, Shizuoka 422-8526, Japan; ^5^Department of Biological Sciences, National University of Singapore, 14 Science Drive 4, Singapore 117543; ^6^South Ehime Fisheries Research Center, Institution for Collaborative Relations, Ehime University, 3 Bunkyo-cho, Matsuyama 790-8577, Japan

## Abstract

Sex chromosomes bearing the sex-determining gene initiate development along the male or female pathway, no matter which sex is determined by XY male or ZW female heterogamety. Sex chromosomes originate from ancient autosomes but evolved rapidly after the acquisition of sex-determining factors which are highly divergent between species. In the heterogametic male system (XY system), the X chromosome is relatively evolutionary silent and maintains most of its ancestral genes, in contrast to its Y counterpart that has evolved rapidly and degenerated. Sex in a teleost fish, the Nile tilapia (*Oreochromis niloticus*), is determined genetically via an XY system, in which an unpaired region is present in the largest chromosome pair. We defined the differences in DNA contents present in this chromosome with a two-color comparative genomic hybridization (CGH) and the random amplified polymorphic DNA (RAPD) approach in XY males. We further identified a syntenic segment within this region that is well conserved in several teleosts. Through comparative genome analysis, this syntenic segment was also shown to be present in mammalian X chromosomes, suggesting a common ancestral origin of vertebrate sex chromosomes.

## 1. Introduction

Understanding the evolution of sex is an on-going challenge for biologists. In spite of the common features of sexual reproduction and associated processes of cell differentiation present in vertebrates, the sex determination systems and sex chromosomes are highly divergent between species and have rapidly evolved [1]. Sex can be determined genetically (genetic sex determination, GSD) and environmentally (environmental sex determination, ESD) in response to stimulation from temperature, other environmental factors, or social cues or a combination of GSD and ESD [2]. Even with the GSD system, different species have adapted diverse GSD factors as a trigger to initiate the onset of sex determination. In mammals, the heterogametic male XY system uses SRY, a sex-determining gene on the Y chromosome (SRY) to initiate the male pathway. In birds and reptiles, a homogametic male or a ZW system uses dosage compensation to initiate the sex differentiation cascade. Thus the initiation factors and the sex chromosome sets are highly variable across species, although some of the downstream regulatory factors driving differentiation may be highly conserved, such as Dmrt1 [3]. In teleost fish, there are a variety of sex determination mechanisms ranging from hermaphroditism to gonochorism and from ESD to GSD [4]. Therefore, teleosts species are especially suitable for studying sex determination from the evolutionary point of view. Different factors initiate the sex-determining pathway among numerous teleost species displaying GSD. For example, a DM-domain transcription factor on the Y chromosome (DMY) has been identified as the master gene in medaka fish (*Oryzias latipes*) [5, 6]; Y-linked anti-Mullerian hormone (*amh*) initiates the onset of sex differentiation in* Odontesthes hatcheri* [7]; and a sex-linked polymorphism of* amh* receptor (*amhr2*) is responsible for male development in* Takifugu rubripes* [8]. Other sex-determining genes include* gsdf*,* sox3,* and* sdY* in* Oryzias luzonensis*,* Oryzias dancena,* and* Oncorhynchus mykiss,* respectively [9–11]. These genetic elements are scattered among various sex chromosomes, with a lack of homology among fish [12] and are not shared by birds or mammals. In tilapia, a group of cichlid fishes that are one of the most important food fishes in the world, few morphological differences exist and few sex-linked molecular markers are available for the cytogenetic identification of X or Y homomorphic sex chromosomes, which are apparently poorly differentiated [13]. Sex can also be easily reversed by temperature, social factors, or hormone treatment in this fish, and most hybrids are fertile [14]. All these features contribute to the reasons why the sex-determining factor and sex chromosome are still poorly understood in this species. Nevertheless, from breeding test [15] in the Nile tilapia (*Oreochromis niloticus*), sex is thought to be determined primarily by an XY system, in which the heterozygous sex is the male (XY). The largest chromosome pair (chr1) has been proposed as the sex chromosome, based on the presence of an unpaired segment in the terminal region in males visualized by synaptonemal complex (SC) analysis [16] and a quantitative sequence difference existing between X and Y detected with DOP PCR probes from chr1 microdissection [17, 18]. Several AFLP markers mapped to chr 1 have been reported to be tightly linked with phenotypic sex in certain families but absent in other families, indicating a distance between these AFLP markers and sex-determining locus [19, 20]. Recently, a single nucleotide polymorphism (SNP) within* amh* was reported to associate to phenotypic male in certain tilapia line [21]. A gene involved in a testicular differentiation, tilapia Dmrt1 (DM related transcriptional factor 1), is not a Y-linked gene as it was largely induced in sex-reversed XX-males where the expression correlates with testicular differentiation [22]. In medaka, phylogenetic analysis revealed that the duplication of autosomal* Dmrt1* and translocation of that copy (*dmrt1bY* or* dmy*) onto the Y chromosome occurred only in certain* Oryzias* species relatively late in evolutionary terms [23]. It is not present in tilapia [24]. So far, seven DM-related genes have been cloned in tilapia, but none of these DM genes are associated with the Y chromosome Guan et al. [22]. On the other hand, estrogen biosynthesis is an evolutionary ancient process and estrogens are indispensable for ovarian differentiation in female fish [25]. The expression of cytochrome P450 (aromatase) which is responsible for production of estrogens from androgens occurs prior to gonad differentiation in female, indicating its potential roles in ovarian differentiation [26]. Aromatase expression was reduced in XX-males produced by hormone treatment or temperature induction [27, 28]. Two types of aromatase genes have been identified in tilapia [29], but neither the brain type nor the ovarian type is located on chr1 [30].

We employed cytogenetic tools and molecular analysis to identify any DNA differences between X and Y chromosomes. Both comparative genomic hybridization (CGH) and random amplified polymorphic DNA (RAPD) assay are powerful methods commonly used to detect molecular differences between normal and cancer cell genomes at the cytogenetic level [31] and to identify sex-linked elements in plants and animals [32, 33], respectively. CGH assay has also been applied in the identification of Y chromosomes in* Drosophila*, the W chromosome in bird, and the Y chromosome in mammals including human [34].

## 2. Materials and Methods

### 2.1. Experimental Animals

Two fish stocks were used in this study. One originated from Egypt and has been inbred for seven generations, termed X^e^X^e^ for females and X^e^Y^e^ for males. The other is Y^f^Y^f^ supermales (Fishgen strain), a commercial strain purchased from the Aquaculture and Aquatic Resources Management program., Asian Institute of Technology in Thailand. All females were produced by artificial fertilization of genetic female (X^e^X^e^) eggs with sperm of neomales (X^e^X^e^) produced by hormonal treatment described previously [28]. All males (X^e^Y^f^) were produced by crossing genetic females (X^e^X^e^) to Y^f^Y^f^ supermales. The X^e^Y^f^ males were mated to X^e^X^e^ females to make an outbred stock. Fish were kept in tanks supplied with recirculated fresh water at 26°C until use. It was confirmed that no sex reversal was observed under these rearing conditions, as phenotypic sex was consistent with gonadal sex, as phenotypic sex was consistent with gonadal sex based on results of sacrificing 100 individuals randomly selected from each experimental batch. Genetic X^e^Y^f^ males utilized in this work consist of five F1 generation individuals (X^e^X^e^ female crossed with Y^f^Y^f^ supermale) and two F4 individuals after three successive generations of X^e^X^e^ and X^e^Y^f^ (sibling-mating).

### 2.2. Chromosome Preparations and Comparative Genomic Hybridization

Metaphase spreads were prepared from head kidney leucocytes of* O. niloticus* according to a previous description with some modification [35]. Leukocytes were collected and mitotically arrested by colchicine (1 *μ*g/mL), prior to suspension in hypotonic 0.075 N KCl. The suspension was then centrifuged and resuspended in Carnoy fixative solution (3 : 1 methanol : acetic acid solution) and then dropped onto glass slides. Slides were kept under −20°C refrigerator until use.

Probes for CGH were derived from genomic DNA extracted from X^e^X^e^ sex-reversed males and Y^f^Y^f^ supermales with phenol chloroform treatment and ethanol precipitation. X-derived and Y-derived probes were labeled with digoxigenin (DIG) or biotin by random priming using “High Prime” DNA labeling kit (Roche), according to the manufacturer's instructions. Hybridization was carried out according to a published protocol [34] with minor modification. Probes were prehybridized separately with corresponding sonicated genomic DNA in 1/100 diluted concentration to eliminate nonspecific binding, background, and noise signals from abundant repetitive sequences present in the genomic DNAs. Thirty microliters of pretreated probe mixture were hybridized to chromosome slides from X^e^X^e^ females and X^e^Y^f^ males in a moist chamber. After 72 hours of incubation at 50°C and subsequent reaction with rhodamine-anti-DIG and FICT-anti-biotin antibodies (Roche Diagnostics, Basel, Switzerland), fluorescence signals were captured by CCD image analysis system (Leica) and documented with Openlab software (Improvision).

### 2.3. Isolation of RAPD DNA Markers

DNA was extracted from adult male (X^e^Y^f^), neomale (X^e^X^e^), and supermale (Y^f^Y^f^) specimens by treatment with proteinase K, followed by phenol-chloroform extraction and ethanol precipitation. DNA mixtures were made by mixing samples from seven males (X^e^Y^f^), eight pseudomales (X^e^X^e^), or two supermales (Y^f^Y^f^). Genomic DNA pools were used as templates to be amplified with PCR reaction mixture containing two arbitrary random decamers (Takara Shuzo, Japan). PCR reactions were carried out with an initial 94°C denaturing step for 3 min, followed by 94°C 1 min, 35°C 1 min, 72°C 1 min for 30 cycles, and a final extension at 72°C for 5 min. PCR products were electrophoresed on 1% agarose gels with ethidium bromide staining and visualized under UV light. Maternal (X^e^X^e^-) or paternal (Y^f^Y^f^-) specific DNA fragments were extracted and subcloned into pGEM-T Easy vector (Promega).

### 2.4. Characterization of RAPD DNA Markers

Southern blotting, STS (sequence tagged site) PCR, and Blast analyses were performed. Four micrograms of genomic DNA samples from males, pseudomales, and supermales were digested with* Hind*III restriction enzyme, subsequently electrophoresed in 0.8% agarose gels and transferred onto a nylon membrane (Hybond-N^+^, Amersham). Hybridization was carried out using probes isolated by RAPD method. Those RAPD fragments yielded a male or female specific restriction fragment length polymorphism (RFLP) and were selected and further validated using STS PCR analysis by designing primers generated from each end of those RAPD fragments. Ten males (X^e^Y^f^) and females (X^e^X^e^) were selected randomly and used to testify the sexual segregation of RAPD markers. Sequences have been confirmed in which amplified fragments from genomic DNA were identical to those from RAPD clones.

## 3. Results

### 3.1. Heterogeneity of Chromosome Pair 1

**Figure 1:**
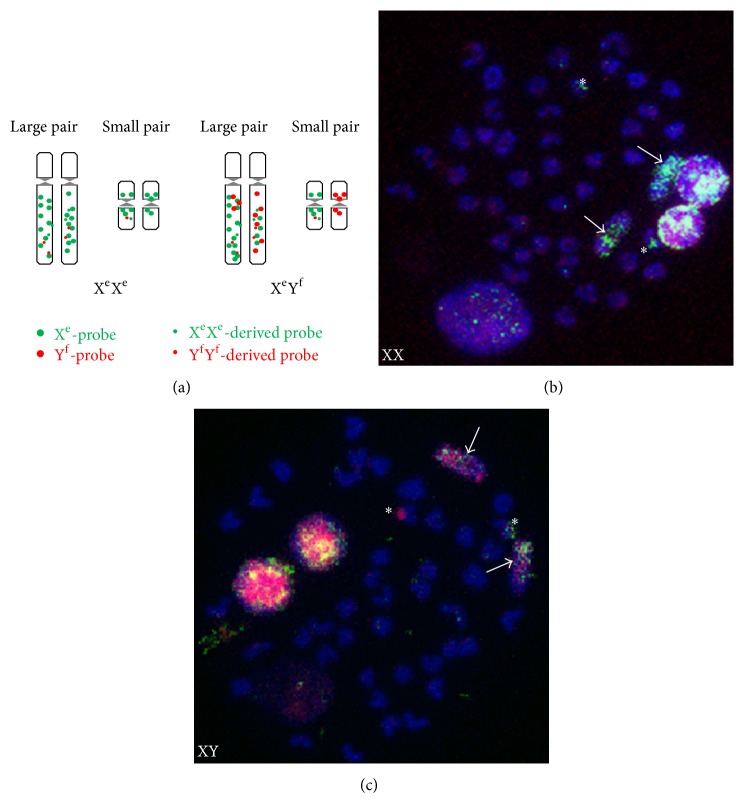
Chromosomal heterogeneity in two pairs of Nile tilapia. (a) Schematic illustration of chromosome heterogeneity detected by two-color CGH analysis. (b) XX metaphase from a female tilapia. (c) XY metaphase from a male tilapia. Signals for XX-derived probe (green) and YY-derived probe (red) are seen on the largest pair of chr 1 (arrows) and a pair of small chromosomes (asterisks).

CGH was carried out on metaphase spreads of X^e^X^e^ and X^e^Y^f^ chromosome with probes illustrated in Figure 1(a). Probes derived from X^e^X^e^ genomic DNA preannealed to the Y^f^Y^f^ sonicated genomic DNA were biotin-labeled and subsequently detected with FITC-anti-biotin antibody (in green) and should identify signals derived from X^e^X^e^ specific DNA contents (Figure 1(a)). In contrast, DIG-labeled probes derived from Y^f^Y^f^ genomic DNA prehybridized to X^e^X^e^ samples were detected by rhodamine-conjugated antibody against DIG (in red). Therefore, using these X^e^X^e^-green and Y^f^Y^f^-red probes, we expected to identify sex chromosome with small DNA content differences resulting from recombination suppression. Results of dual-color CGH are entirely consistent with our expectations. The difference of DNA contents between the largest chromosomes in XY samples was obvious in XY spreads compared to X^e^X^e^ spreads shown in Figures 1(b) and 1(c). Signals from the Y-derived probe hybridized preferentially at the long arm of chromosome 1 (chr 1), the largest sex chromosome, in contrast to the X-derived signals which were evenly distributed over the whole region of this chromosome. Thus, a difference was clearly visualized by this dual-color CGH in XY spreads. In addition to chr 1, the DNA heterogeneity extends to one small pair of chromosomes identified by asterisks in Figures 1(b) and 1(c), indicating some difference between X^e^X^e^ and Y^f^Y^f^ strains, which may be linked to phenotypic male and female sex.

### 3.2. Isolation of a Putative X-Linked Marker

**Figure 2:**
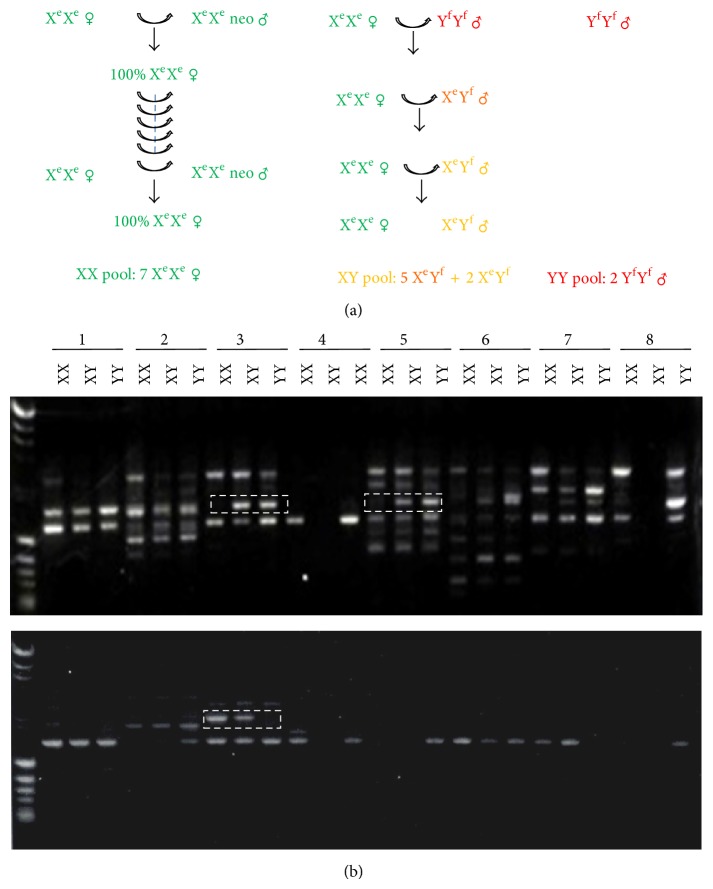
Strategy of RAPD sex-linked marker selection. (a) DNA pools are derived from XX, XY, and YY individuals. (b) RAPD PCR was performed with random primer sets. Fragments derived from X-linked or Y-linked are in dashed box.

**Figure 3:**
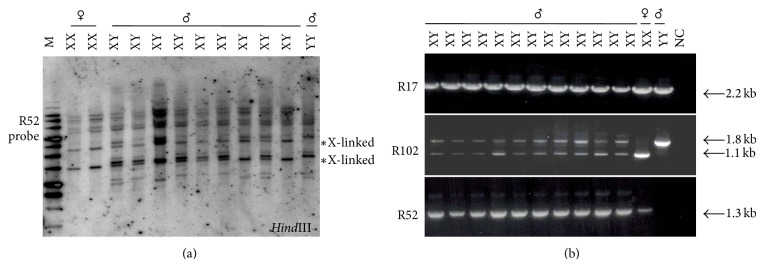
Characterization of RAPD markers. (a) Southern analysis revealed two X-linked fragments present in XX females and XY males but absent in YY males. (b) Results of STS PCR with primers derived from R17, R52, and R102 are listed in Table 1. An X-linked 1.3 kb band was only amplified with R52 primer1 and 2 with DNA from XX female and XY males, in contrast to a maternal 1.1 kb and a paternal 1.8 kb fragment in R102 and a 2.2 kb fragment of R17 from all XY, XX, and YY autosome. NC: negative control with H_2_O instead of DNA as a PCR template.

A total of 298 primer combinations from 24 arbitrary 10-mer oligonucleotides were tested using DNA pools from eight X^e^X^e^ pseudomales, seven X^e^Y^f^ males (five F1 X^e^Y^f^ male and two F4 X^e^Y^f^ males after three successive generations of X^e^X^e^ female mating to X^e^Y^f^ male), and two Y^f^Y^f^ supermales, respectively, as shown in Figure 2(a). Products from sixteen primer sets displayed a specific fragment derived from X^e^X^e^ and/or Y^f^Y^f^, with an example shown in Figure 2(b). Polymorphisms from ten primer combinations were replicated by PCR using DNA from each individual fish. Eleven fragments were finally excised, gel purified, and subcloned for further analysis. Five clones were sequenced for each excised fragment. Clones present more than twice among each five sequences were used as a probe for Southern analysis and further confirmed by a more reliable and reproducible STS-PCR analysis. Two X-linked fragments were detected from RFLP analysis with probe for R52, indicating that R52 was associated with the X chromosome (Figure 3(a)). Putative sex-linked bands were also found with probes of R17 and R102 (data not shown). Primers were designed from both ends of putative sex associated fragments for STS-PCR. The R17 probe revealed a polymorphism unrelated to genotypic sex (Figure 3(b) and Table 2). The R102 probe revealed an additional band derived from Y^f^Y^f^ individuals; this polymorphic fragment was inherited in all X^e^Y^f^ (*n* = 87) specimens but was not seen in F4 X^e^Y^f^ males (*n* = 10) after three successive sibling matings, suggesting that R102 is not associated with the Y chromosome. Primers derived from R52 amplified a fragment in X^e^X^e^ females and X^e^Y^f^ and X^e^Y^e^ males but not Y^f^Y^f^ (*n* = 6) supermales, indicating that R52 is X-linked (Figure 3(b)).

### 3.3. Comparative Genome Analysis of R52

**Figure 4:**
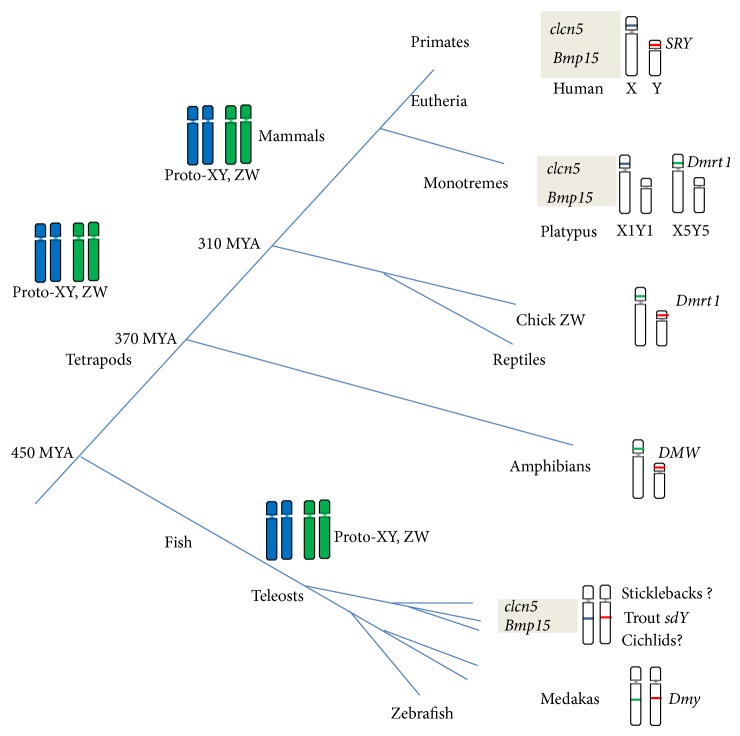
Processes of syntenic segment addition or transmission during sex chromosome evolution. (blue) *▬*, (green) *▬* Relic of protosex chromosomes; (red) *▬* male specific regions on the Y (MSY). Divergence times were from the literature [58, 59].

The tilapia genome has recently been sequenced and partially annotated into the genome database Orenil1.1 (http://www.ensembl.org/index.html), which allows for positioning of any sequences into tilapia genome. R52 was localized within the putative sex-determining region on chr 1, between the scaffolds GL831391 and GL831276, which harbor two sex-linked markers UNH115 and GM180 (Table 2) in respect [36, 37]. Evaluation of genome conservation between rainbow trout and three teleost models, medaka, stickleback, and zebrafish, provides evidence of syntenic segment conservation in certain teleosts [38]. Colocalization with* sdY*, the sex determinant in sex chromosomes of rainbow trout [10] and the putative sex chromosome in the stickleback VII group [38] led us to undertake comparative mapping from fish to mammals (Figure 4), assuming that their sex chromosomes are from the same ancestor across phyla. We found that this region including chloride channel protein 5 (*clcn5)* is highly conserved in X1 chromosomes of mammalian monotremes such as platypus and also in the PAR region of the X chromosome of therian mammals, indicating a previously unrecognized evolutionary link from fish to mammals [39].

## 4. Discussion

We employed CGH to visualize the DNA difference on chr 1, the poorly differentiated sex chromosome in Nile tilapia. We further isolated an X-linked DNA marker R52 that is localized in the putative sex-determining region on chr 1. Sex chromosomes are thought to be derived from chromosome pairs which were originally homologous. Differences between X-Y and Z-W result from the suppression of meiotic recombination in a region flanking the sex-determining genes. Due to the accumulation of genetic changes, sexual selection, and genetic drift, these differences increased over time and led sex chromosomes to vary in morphology and in DNA contents. It has been demonstrated that CGH is capable of identifying the differentiated sex chromosomes in both the XY and ZW systems of mammals and insects, respectively [34]. Highly sensitive dual-color CGH enables us to successfully identify differences spanning a large region on the long arm of chr 1 in XY males, corresponding to the findings from SG analysis and chromosome painting above. Using dual-color CGH, we confirmed differences of DNA contents in the putative sex chromosomes in Nile tilapia, in which Carrasco et al. [16] reported an incompletely paired segment present in the largest chromosome pair (chr 1) of the heterogametic genotype during the process of meiotic synapsis, with nucleotide diversity occurring in this unpaired region. This nucleotide diversity was also detected by chromosome painting with probes derived from chr 1 microdissection [18]. In addition to chr1, a small pair of chromosomes also displayed diversity in XY male mitotic metaphase in our CGH analysis, indicating that the nucleotide diversity between male and female also exists on this small chromosome pair, although strain-dependent sequence heterogeneity should not be excluded. This raises the possibility of multiple chromosome sets controlling sex determination in Nile tilapia. Multiple loci and multiple sex chromosome sets involved in directing testicular differentiation in tilapia were proposed from bulked segregant analysis [36] and sex ratio departures of Y^f^Y^f^ male progenies [19]. Two sets of incompletely paired chromosomes (both the biggest chromosome and a small one) were observed by SC analysis in* O. aureus*, the species closely related to* Oreochromis niloticus* [40]. Although* O. niloticus* is proposed to display male heterogamety, in contrast to female heterogamety in* O. aureus,* they belong to the same genus. They share several similarities as both have similar mitotic karyotypes in chromosome number (2*n* = 44) and morphology. Probes developed from chr 1 in* O. niloticus* also cross-hybridized to the chr 1 of* O. aureus* [41], indicating the existence of homology between these chromosomes. The restriction of meiotic recombination to the small chromosome of* O. niloticus* may be undetectable due to the limited resolution of SC analysis but captured by the more sensitive dual-color CGH analysis.

RAPD is a sensitive method to resolve differences between male and female genomes. We isolated one putative sex-associated DNA markers in tilapia with RAPD method. STS PCR using primers derived from R52 identified a putative X-linked allele in two experimental stocks that originated from different genetic backgrounds. From a total of 298 combinations of primer sets, only one RAPD marker was isolated to be putatively sex-linked, reflecting a very slight difference between male and female sex chromosomes, which was corresponding to the homomorphic and poorly differentiated sex chromosome sets in tilapia. The X-linked R52 marker is assembled and colocalized in a highly conserved region of LG3 containing UNH115,* clcn5* gene, and GM180 [42]. Both UNH115 and clcn5 were physically mapped to chr 1 within a putative sex-determining region [13, 43]. Notably, they are annotated on LG16-21 according to the genome database rather than LG3 demonstrated by genetic mapping and physical mapping with FISH. The result of a LG16-21 location derived from Orenil1.1 blast could be considered either from errors in Orenil1.1 database assembly or strain variation in chromosome structure among strains (personnel communication from T. Kocher).

Comparative genomics of conserved synteny across phyla facilitates tracing the origins of protochromosomes prior to their reshaping and reconstitution. It also enables the discovery of events and mechanisms occurring in vertebrate genomes across large time scales during evolution. Sex chromosomes have been assumed to have evolved independently during the evolution [39, 44], with different species adapting different ancestral autosomes as their protosex chromosomes. For instance, few signs of ortholog are found among sex chromosomes of human (X or Y) and birds (Z or W) [45]. Some particular chromosomes are repeatedly used as sex chromosomes in several teleost fish [46]. In fact, the syntenic segment of* clcn5* within the putative SD region in tilapia is also found in the sex chromosome of rainbow trout, where the Y-linked* sdY* localizes [10] and in* stickleback*, revealing a common segment shared among these sex chromosomes, indicating that they are derived from a common ancestor. This syntenic segment is further well conserved in X1, one of multiple sex chromosomes in platypus. Homology of platypus X1 and X5 with the therian (eutherians and marsupials) XY PAR and bird ZW-system, respectively, is consistent with this species' position in the phylogenetic tree between birds, reptiles, and therians [47]. Evidence of the presence of this segment in eutherians and human X chromosome's pseudoautosomal regions (PARs) [48] supports a hypothesis of a common ancestor of certain teleost fish evolutionarily linked to mammal lineage. Interestingly, medaka sex chr 1 is related to bird ZW and platypus X5Y5 and contains the Dmy/Dmrt1by gene [6, 49], consistent with the idea that protosex chromosomes arose independently in medaka and mammals [12, 47, 50]. Nevertheless, sex chromosome sets of the monotreme show homology with the therian XY system at one end and to the bird ZW system at the other end, thus providing an evolutionary link between XY and ZW systems that were previously thought to be independent and unrelated [51, 52]. Evidence from DNA markers from mammalian X and bird Z syntenic in a salamander (*Ambystoma*) [53], suggests that these regions are evolutionarily related. We do not know whether this homology is a relic of shared ancestry or merely that a specific transposable element is favored for sex chromosome acquisition. However, our striking findings of this link in X chromosomes from fish to mammals should provide insights for understanding the process of sex chromosome evolution in vertebrates.

## 5. Conclusions

Production of genetic males is a common way to improve the production efficiency in tilapia aquiculture. This can be achieved via several approaches such as interspecific hybridization [54], hormone treatment [55], and YY supermales [15]. Production requires several generations to establish the YY supermale stocks. Our RAPD markers together with other sex-linked DNA markers [56, 57] will help to simplify the process of YY supermale production and screening of individuals as males and reduce the costs in commercial aquaculture of tilapia. These sex-linked markers are also indispensable for the study of sex determination loci in tilapia and will be useful in understanding the mechanisms of sex determination in low vertebrates, as well as sex chromosome evolution in vertebrates.

*Accession Numbers.* Sequence data from this paper have been deposited in the GenBank (http://www.ncbi.nlm.nih.gov/Genbank) under accession numbers R17: DQ323031; R102: DQ334865; R52: DQ351276.

## Supplementary Material

Figure S1: sequence of R52 (DQ351276) The STS PCR primer site is indicated in bold and highlighted.
Figure S2: sequence of R17 (DQ323031) The STS PCR primer site is indicated in bold and highlighted.
Figure S3: sequence of R102 (DQ334865) The STS PCR primer site is indicated in bold and highlighted. Sequence derived in blue is associated with Y^f^Y^f^ strain, not related to phenotypic sex.

## Figures and Tables

**Table 1:** Sequences of RAPD clones and primer sets for STS PCR.

Name	Size (kb)	Accession	Primers
R17	2.2	DQ323031	R17F: GATCCAGTACCACAAGGC R17R: GATCCAGTACCTCGAGACC
R52	1.3	DQ351276	R52F: GGAACCAATCACAGGTAAGG R52R: GATCAAGTCCTTACATGTGG
R102	1.8	DQ334865	R102F: GGAACCAATCTTTGCAACAG R102R: GGAACCAATCAGGACACGTT

**Table 2:** High identity present in region among UNH115-R52-clcn5.

Marker	Accession	Scaffold (position)	Linkage	Identity (%)	Length (bp)
UNH115	G12268	GL831391 (20192–20477)	UNK70	96.27	295

R52	DQ351276 (1120–1304)	GL831391 (883646–883824)	UNK70	94.59	185
	(840–988)	GL831391 (882411–882562)	UNK70	89.47	152
	(679–871)	GL831276 (180605–180797)	LG16-21	92.23	193
	(501–677)	GL831276 (180800–180976)	LG16-21	80	177
	(256–392)	GL831276	LG16-21	89.78	137

*clcn5 *	DU133417	GL831276: 644643–645242	LG16-21	99.83	600
