# Supplementary material for: A Syntenic Region Conserved from Fish to Mammalian X Chromosome
Source: Int J Evol Biol. 2014 Nov 18;2014:873935. doi: 10.1155/2014/873935 (PMC4254068; doi:10.1155/2014/873935)
Supplement: Supplementary file 1 — Figure S1: sequence of R52 (DQ351276) The STS PCR primer site is indicated in bold and highlighted. Figure S2: sequence of R17 (DQ323031) The STS PCR primer site is indicated in bold and highlighted. Figure S3: sequence of R102 (DQ334865) The STS PCR primer site is indicated in bold and highlighted. Sequence derived in blue is associated with YfYf strain, not related to phenotypic sex. [file 873935.f1.pdf]

```
       R52F→
1      GGAACCAATCACAGGTAAGGACTGAAATCTTCTAGCTGTTGTTACAGAAAAGTGCTTTCACCAAGAACATTAATGGATGT
81     GACCAGCAGCATGTGTTCAACAACATATAGCTATGGTCACTTAGAATCATCTATTAAACATATGCTTAAGCGTAAACTTT
161    CTGTTGTTAGAAATAAGGGTGAGTTCTTGTTCTTGTTTACTTTTTATATCTTTTTCATTTGTGTTTTTTTTTTTTCTTTGC
241    TGTGAATCATTTTTATCATTCACTAGAAAATGATCATTTTAAAACCACAATTTTCCTCCACAGACGTTTCTTCATGTCCA
321    ACAACAACTCCTGGTATGTGAACATTCTTCTTCTTTCCTTGTTTATTCTTTAAAGTATATTTTTGCAGTAAAAGTCCTTA
401    AAAACTGTGTAGTGATTGTGCTTTTAAACTTTTTTTTTTTTTTTTCAACATTTCACTTTGTGTTAATAGTTTATATAGGTT
481    CAAATATACATTTAAGCTGTCTTTTATGACCAAGGCTTATACAGTGATAATATGTAACCACAGCACTTGAAGTGCTTTGC
561    TGCATCTCCTTAAAGGAAACACTTGGATATTAAAGACCTAATAANGNAATTTTGCAAGCTTGGATTGCATTAAATCCTGN
641    GGCTGCATTTGAAACAAATAATTTGAATTTTAAAAAANGTATGTGGAATTTTAAACTGNTGCTTTTTAACCCTTTTTCCC
721    ATAGAAATAACACCNTCACTTACACCAACATNCAGTAAGACGTTTGAAATGTATAGTGAAGAATGAAGGTCTTTGTGTGA
801    GCTCCAGATTATCAGTTGCACTGTCAAATCTAAAAAAATGAATCACTTTTGTCTCAACAATACAGTTCTTTAAATGATCT
881    AGAATCACACGTGTTTGGCTTGTGACTCAATTAGCTGAGATTATTTCAACTTTCAGTTTAAGTGGTAGTAAATAAGGTTT
961    GACAGTACCAGATTAGAACAGTGTGGACGTGGGGAGCAGATGCAGCATCAAAATGTTGACTATGTGGAAACGGGCCATAA
1041   AAGTGACAGATTTTGTCAGCATTTTCTTGGAAATATCATCTCCCATCATTGTGTTATGTATGTGATGGATTTGCCCCCTT
1121   TTTCTCCTTCACAGCACCAATGGACTTTGATATAGTCCTTGAATTAAACATCCCAGCATCCAGTGTGGCCCCAGATTTCA
1201   GTGATGTACTCAGACACATTGTGAGAAATGTCTCCCTAACCCAAATCATCACCGAGTCTTTAATACTGAAAAACCTGACC
1281   TTCACCACATGTAAGGACTTGATC
           ←R52R
```

Fig. S1 sequence of R52 (DQ351276) The STS PCR primer site is indicated in bold and highlighted.

```
        R17F→
1       GATCCAGTACCACAAGGCCGTATGAGTTCACACTTTTTACTCACAAGAAAAGTGTGAGTCATCGCAGGGATTTTATGGAG
81      ATATTAACTTCACATGGCGCTGGTGGACCTTTAAGTCATTATGATGATAAACCCCACAAACACACTTTTATCTGATTTTT
161     AAAGACTGAACAATCAAAAAAGGATCACTTAAGCAGTCTCCCATTTTTAGGTAACAAATGAGCATATTTGGATGTCTTCT
241     ACAACACTAGAGTAGATTTGAAACAACCCCATCACAACTCTCAAACTAAGCCGAGTACATCTAAAGTTAGTTTTGTGATC
321     TGCGTTAATGTAAGTAACAGTGAAAACGGCATAAATAAGCACAAGTCACAGCTGGTAGGAATTTATGCAAACATTAACAG
401     ACCTAATTAATAAATAACAAGATACATACACCTGCTAAAGCACGAAAAATCTCCGCAGACTATGGATCACGAAAAGATAT
481     CTGCCTTTGTTTGTGTTATTTCCTTATGAACACTAAATGATTCCTCTCCATTTTTTCTGATTATAATTTGTTATGCATTT
561     TTACGAAGATGCAAAGCTAAATGTCAAGTTTCACCCTATCTCACGGTGGAAAAGAATTCTTTCAAACATCAAAGATGCTT
641     GGCTCCAAATGTTGGACGCACTGATCAATCCTGCACACAAATACAAAAAATTAACTAATCCCATACCACTGTGACAAATC
721     AACCTAACAGTGATGACCAAATGATTGCCTCATGAAATAAAAAAATATAGGTAAAATAGAAATATCTGAAAGTCCCCTGC
801     ACCTCTGACTTTTAAGATGTACCACGTCTAAAATACAAACATACAGCATTTATTTAGCATTGAGCATTCAGTATTAACAT
881     TGGCAGTACATGATTATGCCACATGTGTTTATTTTTATTGTAAACTTGCATTTATATTAACTGTCTCATTATCAATATGC
961     CGTGTACAAATATCTTTACTTGTCCCACTCAAAATGATCTAATGAGTACTAATAGGTTTTTACACCTCCACAATGAGGCG
1041    ATTCCTGTCTATCATGATAAATAATCAGCAGTTGGTGGTTCACAAAATAGATCTTCTTTCACAGCCGCAGCCTTATTTAT
1121    TTTTGTTTTGTTTTCTAAAACATGGAAACCCAGTTAAAATGTCAGGGCTGTGTTTATGATAGAAAATAAGTCAAGTTTAT
1201    AGCAAGGATTTATTCAACAGTTCACAGGCAAACAGAGAAAGCAAGAAACCCTATGTACCACAAAATAGACAGCCAACCTA
1281    GTTTCACAAGAGCTTTAAATTAAAATGCTGTTTTACCCATAGGCCACTGAGGGATTTTACCACGGTGCTAGTAGAATGGT
1361    GAAGAAAAAAAAATTCCAAGAGTCATCTAAAGGAAAAAAAGCGCTGTGTGCATTAGGCGATATAGAACTAATTCAGCCCAC
1441    CCTTTCCACCCTAGAGCTATATAACAGCTCAGAGGTAAGTAAACCAACACAAAACCTTCTCTAAACAACTTGCTCTTCAC
1521    ACTGACAAATCTTCAAGCTGAATATGAAGCACTTTATTAGGATTGCCTTTTGGATTGTTACTATTGCTGTTTTTTGCTGG
1601    AACACTCAAGGGAAACCTATATCCAAACCCTTAATAGAAATGAGTGACTTGACACTTTTAAAGAACGTCAGCTGTGTAAG
1681    TATCAAGATGATTTTTTTAACGGGTTTGTAAACTATCTGCTATGTGCTAATAAATACAATGTCTTCTTCTATATTTACAG
1761    ATTGACGGTTCCGAATTCTATGCTCCTGAAAATATAGAGGTAAGACTATATTTAAAAACTTGCATTTACATTTCTTGAAA
1841    AATGTAGTGCTGCCAGAGACTTCTGTCCGTCACAAATAAAAATAAACTGTATAGCCACTGTACCAAACAGAAGATAATGA
1921    TGATAGGCTGATCAGGGAATAAGAAGTCAGAACAGAAGGTGTGATGTTGGGTAGTCCCCATATGCTAGTTAAGCTTTTAT
2001    AAACCACATGTAAAGGTTCTCTAGTACAGGAAATGAGCTTGCCAGGTGTCGATATTTCACACAGCTGAGCCTGCCTCTAT
2081    TTGCAGCCACAAGCCTGTCTCAGAGCAGTAAAAGAAAAATCTTTCTCTGCCACACAGAAAGAATGCTTCATTAACGCACT
2161    GAACTGCGTTACCTCAGAACTTGAAAAGGCAAACAAGTCAGGGGAATGTCGAGGCTCAGAGGAACGCATTCTCCAGGGTC
2241    TCGAGGTACTGGATC
             ←R17R
```

Fig. S2 sequence of R17 (DQ323031) The STS PCR primer site is indicated in bold and highlighted.

	R102F➔
1	**GGAACCAATCTTTGCAACAG**AGGCACAGAGGAGAGACAGGAGTCTACTGCCACTTTAAATTTTTTAAATCATTGAGGAAA
81	AAGACCCTCAAAGTCAATCTGAGTGGGTTTGGATTTCTTATTTTATCCAAGCAACAATCCAAAACCAATAGAAAAAGAGT
161	CCAAAAACCAGAGAAAGGTTTGCATGAAAAATGACAGAAACTATGGGGAATTTCATATACATATTTTCCTAATGCTCTAA
241	CCTTGATGGTTTCACAGCCTATTAACCCTTATCACCAATTAACAAGGCCTAAAACTGTCATTTCTGTCTTACACCTTTAC
321	ACCTGATAATAAACATCCACACCCTACTGAGAAAAAGCTCTCCAGGTCAAAGAGGTACCTCTTCTGATGGTGTTAGGGGA
401	AGGAGACAACCAACCTTTACTGAGACATTGGTGCGGCCCACTTCACTTTAAATGGTTGAAGGCAAGGCGGCCAGAATAAG
481	TGGCAAAAAAATGTTATTTCAAAGTCCAAAGCTTTTACAACTGACTGACTGCAAGATAAATAAAGTCGATTCAGGAGCAG
561	TGTATCTGAACATTTTCATCCATCCAGTCATGGCTGTCGTTAGCACTTTAACCTAAAAAAGTCCGGCTGCCTTAAGACTA
641	AAAACAGTGCTACTACTTTTAATAAAGGGAATAATAAAGGAAAACTGAAGGACTTTAACCAAAGAGTAGCATCAATGCAA
721	TAGACGCATGTTTAAGATAAGATAAGATAAGATAAGATGGCCTTTATTAGTCCCACAGGTGGGAAATTTGTTTTGTTACA
801	GCAAAAGTGCAAAGTTATGTAGCAGAAATTAGAAAACACTGGAATGCAATAAAATACAATAAAATAAAATAAAATACTAT
881	ATACAATAGAATAAAATAGAATAGAATAATATATACAATAGAATAAAATAGAAATACAAATACTATATACAACTGAGTAG
961	GAAAATACAAAAACACAACTTCGTCAGAAAGAGAATTGCACGTATAGCAGTCTTATTGCACATGTGTGGGTTTGTGTGTT
1041	TGATCAGCTGCAAAAGTCTTTATTGTAGAGTCTGACAGCAGTGGGGAGGAAAGACCTGCGAAATCTCTCCGTCCCACACC
1121	GTGGGTGCCGCAGTCTCCCACTGAAGGAGCTGCTCAGTGCCTTCACAGTCTCATGCATGGGGTGGGAGATGTTGTCCAAC
1201	AGGGATGACAGCTTAGCCACCATTCTCCTGTCACTCACCACCTCCACTGGGTCCAGAGGGCATCCTAGAACAGAGCTGGC
1281	CCTTCGGATCAGCCTGTTCAGTCTCTTCCTGTCCCCAGCAGAGATGCTGCCCCCCCAGCAGACCACACCATAAAAGATGG
1361	CTGAGGCCACCACAGAGTCATAGAAGGTCTTCAGGAGTGGGCCCTCCACTCCAAACGACCTGAGTCTCCGAAGCAGGTAC
1441	AGCCTGCTCTGCCCTTTCCTGTAGAGGGCGTCTGAGTTATGAGTCCAGTCCAGTTTGTTGTTCAGATGAACACCAAGGTA
1521	CCTGTAGCTGTCCACAGCCTCGATGTCCATACCTTGGATGTTCAGTGGTTGCAGTGGAGGATGTTTGTGCCTGCGGAAGT
1601	CTACCACCAGCTCCTTGGTTTTACTGGCGTTGATCTGGAGGTAGTTCAGCTGGCACCAGTCCACTTTTTAAAATGGACTG
1681	GTAATACTTTTTAAAATGTATTAAAAAGGCCTTTTTTTCTGCAGCATAGTTT**AACGTGTCCTGATTGGTTCC**
	🡐R102R

Fig. S3 sequence of R102 (DQ334865) The STS PCR primer site is indicated in bold and highlighted. Sequence derived in blue is associated with Y^fY^f strain, not related to phenotypic sex.
